# Assessment of Barriers to Knowledge Sharing Among Medical Students in Riyadh, Saudi Arabia

**DOI:** 10.7759/cureus.45665

**Published:** 2023-09-21

**Authors:** Zeeshan Feroz, Abdulelah Alghamdi, Ismail Memon, Khaled A Alhabdan, Faisal Almehrij, Bandar Alshahrani, Mohammed Abanmi

**Affiliations:** 1 Basic Sciences, College of Science and Health Professions, King Saud Bin Abdulaziz University for Health Sciences, Riyadh, SAU; 2 King Abdullah International Medical Research Center, National Guards Health Affairs, Riyadh, SAU; 3 College of Medicine, King Saud Bin Abdulaziz University for Health Sciences, Riyadh, SAU; 4 Anatomy, Saba University School of Medicine, Saba, SXM

**Keywords:** riyadh, medical colleges, medical students, knowledge sharing, barriers

## Abstract

Background

Knowledge sharing is a process by which information is exchanged between peers, colleagues, or, at a higher level, between institutions and organizations. This study aimed to assess the barriers to knowledge sharing among medical students at private and public-sector medical colleges in Riyadh, Saudi Arabia.

Methodology

An online questionnaire was used to collect data from four medical colleges. Students were selected by non-probability convenience sampling. The English-language questionnaire included 12 questions related to knowledge sharing based on a Likert scale of one to five, with one denoting strongly disagreeing and five strongly agreeing. Out of the 520 questionnaires, 497 (96%) were received and analyzed using SPSS version 23 (IBM Corp., Armonk, NY, USA).

Results

A total of 497 respondents completed our questionnaire. Most were males (67.8%). Our results revealed that statements such as “afraid to provide the wrong information,” “people only share with those who share with them,” and “too busy/lack of time” were the most perceived barriers to knowledge sharing (mean = 3.95, 3.61, and 3.60, respectively). Furthermore, female opinions on statements such as “lack of relationship,” “afraid to provide the wrong information,” “do not know what to share,” and “shyness to provide own opinions” were more dominant than male opinions. This difference was found to be statistically significant (p-values = 0.007, 0.020, 0.002, and 0.009, respectively).

Conclusions

Our study indicated that barriers such as “afraid to provide the wrong information” and “people only share with those who share with them” are important barriers that hinder the process of knowledge sharing. Moreover, most students agreed that “too busy/lack of time” and “lack of relationship” are barriers to knowledge sharing. In addition, statements such as “lack of relationship,” “afraid to provide the wrong information,” “do not know what to share,” and “shyness to provide own opinions” were acknowledged as barriers by female students more than male students. There is a need in the curriculum to structure various types of activities that inspire and promote knowledge exchange among students. Further research is needed to validate our findings.

## Introduction

Knowledge can be described as the comprehension, insights, and abilities that one acquires as a result of education or personal experiences. Knowledge sharing is a process by which information is exchanged between peers, colleagues, or, at a higher level, between institutions and organizations [1]. Knowledge is now considered the key asset in institutions; the management of that knowledge is important to guarantee the success of institutions’ progression in many different aspects [2]. Clinical and basic medical sciences, along with other aspects, are considered enablers for achieving the best medical outcomes in terms of patient care. Clinical and basic medical knowledge can be acquired in various ways. One is through academic activities conducted in medical colleges, which include lectures, case discussions, and lab work. Another way of acquiring this knowledge is through the comprehensive reading of validated medical references, such as books and research papers in the medical field. Certain patterns have been recognized where some people retain information to gain a higher rank or position, or, in the case of students, to gain a higher mark. Furthermore, as individuals cannot be forced to share knowledge, they must be encouraged and influenced to share their knowledge with their peers. Although some want to share knowledge, unfortunately, several factors, such as a lack of support, encouragement, and a knowledge-sharing culture, may hinder the process of sharing knowledge.

Various studies have been conducted to assess the factors interfering with sharing knowledge. Most of the studies aimed to assess the attitude of employees working in organizations regarding knowledge sharing to gain higher efficiency and efficacy [3-7]. Globally, limited research efforts have been made to identify these impeding factors among medical students, as only one study discussed this specific dilemma. A study conducted at the University of Lahore in Pakistan focused on evaluating the barriers facing medical students in college that interfered with the knowledge-sharing process [8]. Similar research has been conducted in Saudi Arabia and some Gulf countries to evaluate the behavior of knowledge sharing among employees working in organizations and identify factors that might interfere with this process. However, no research has been conducted to assess these factors among students in academic and high-level education [9-12]. This reflects a lack of awareness of the importance of sharing knowledge and how such a process could add enormous value to the learning environment in medical colleges and universities. It also reflects the lack of awareness about how students can join hospitals as better doctors by identifying these obstacles.

As medical students ultimately join the healthcare system and become physicians, it is important to identify the factors hindering the knowledge-sharing process among them to obtain better learning outcomes in medical colleges. As the medical field is highly competitive, especially for students, barriers that limit the sharing of information are easily created among students. By identifying these barriers, we can help other medical educational institutes eliminate them. This will create a thriving, successful environment for medical students to share their knowledge with their peers and colleagues. Helping medical students to share knowledge freely will not only help them to gain higher and better grades but will also pave the way for them to become better physicians, where they will add great value and make improvements to the medical system.

Knowledge sharing can be significantly influenced by religious or cultural beliefs. Different communication styles, levels of transparency among societies, contextual relevance, and other factors are capable of having a direct effect on how people share knowledge. This study aims to explore knowledge-sharing attitudes and identify the potential barriers among medical students. By identifying these barriers, medical college administrations will know how to empower students to gain better educational outcomes and create a healthier learning environment among them. The objectives of the study include assessing barriers to knowledge sharing among medical students in medical colleges in Riyadh, Saudi Arabia.

## Materials and methods

Study design

The participants in this study were from four leading medical colleges in Riyadh, the capital of Saudi Arabia. These medical colleges belonged to King Saud Bin Abdulaziz University for Health Sciences (KSAU-HS), King Saud University (KSU), Alfaisal University, and Imam Mohammed bin Saud Islamic University (IMSIU). In this cross-sectional study, non-probability convenience sampling was used to include medical students who were available and willing to participate at the time of data collection.

Identification of study participants

The target population was the medical students studying at the selected universities. The approximate number of students in these colleges was around 5,000.

Inclusion and exclusion criteria

All male and female students enrolled in the selected medical colleges were considered for inclusion in this study. Students in the preparatory year were excluded.

Sample size

The estimated sample size was around 500 students currently studying in medical schools. The response distribution was kept at 50% as no previous study has estimated the prevalence of barriers to knowledge sharing among Saudi students. With a confidence level of 97% and a margin of error of 5%, and by using a Raosoft sample size calculator, the sample size was calculated at 427 students from four different universities. The sample size that we achieved was 497 participants. Most of the study population was from KSAU-HS, with 231 participants, followed by 146 from KSU. In third place was IMSIU with 76 participants, and the least studied population was from Alfaisal University with 44 participants.

Data collection process

This study was questionnaire-based. The questionnaire was adopted from a previously published study [8]. The reliability of the questionnaire was assessed using Cronbach’s alpha with a value of 0.68. The questionnaire was validated in a previously conducted study, and the same questionnaire was used with the same elements and in the same language. Data were collected through an online questionnaire distributed through the official university email of the targeted population. The questionnaire was in the English language, and medical students were able to respond in English. The first section was related to the demographic profile, including variables such as age, gender, current year of study, the name of the university, and type of university (public or private). There were 12 questions related to knowledge sharing; 11 of them were fixed barriers to be answered on a Likert scale ranging from 1 to 5, with 1 being strongly disagree and 5 being strongly agree. The last element of the questionnaire was left blank under *others* to allow the recipients to share any other barriers from their own perspective that were not included in the 11 fixed barriers of the questionnaire. However, no responses were received from the studied population regarding other barriers not mentioned in the questionnaire. The total score of the questions was used. The maximum score was 60, while the minimum was 12. Knowledge sharing was the main outcome variable.

Data analysis

A total of 520 responses were received, of which 497 responses were complete and 23 were incomplete. Incomplete responses were eliminated from data analysis. The collected data were entered in Microsoft Excel version 2019 (Microsoft Corp., Redmond, WA, USA). SPSS version 23 (IBM Corp., Armonk, NY, USA) was used for data analysis. Descriptive statistics were reported initially, and categorical variables such as gender, year of study, and name of university were reported as frequencies and percentages. Numerical variables such as age and hours of study per day were reported as mean and standard deviation (SD). The total score for the items was computed and initially reported as the mean ± SD. Independent-sample t-test and one-way analysis of variance (ANOVA) were used to assess the relationship between knowledge sharing and demographic variables such as gender, year of study, and type of university. For all the tests applied, a p-value of 0.05 was considered significant. Statistical tables were used to present the statistics.

Ethical considerations

A consent form was attached to the questionnaire, and participation was entirely optional. Participants had the right to withdraw at any time, and no personal information regarding the participants’ identification was required. All collected data were kept confidential. This research project was approved by the Institutional Review Board (IRB), King Abdullah International Medical Research Center (KAIMRC), Riyadh, Saudi Arabia (study number: SP20/334/R; Memo. Ref. No.: IRBC/1491/20, E-CTS Ref. No. RYD-20-419812-104352; approval date: August 31, 2020).

## Results

Sociodemographic characteristics of study participants

This study included a total of 497 respondents. In total, 337 (67.8%) of the participants were male. KSAU-HS students represented the highest proportion of the study population with 231 (46.5%) participants, followed by students from KSU with 146 (29.4%) participants. Regarding students’ current year of study, we found that more than one-third (36.6%) of the participants were third-year students, followed by first-year (25.4%), fourth-year (16.3%), second-year (12.7%), and fifth-year (9.1%) students. First-year students accounted for one-fourth of the study population (Table 1).

**Table 1:** Sociodemographic characteristics of study participants (n = 497). KSAU-HS = King Saud Bin Abdulaziz University for Health Sciences; KSU = King Saud University; IMSIU = Imam Mohammed bin Saud Islamic University

Variable	Frequency	Percentage
Gender		
Male	337	67.8%
Female	160	32.2%
University		
KSAU-HS	231	46.5%
KSU	146	29.4%
Alfaisal University	44	8.9%
IMSIU	76	15.3%
Current year of study		
First year	126	25.4%
Second year	63	12.7%
Third year	182	36.6%
Fourth year	81	16.3%
Fifth year	45	9.1%

Barriers to knowledge sharing among medical students

This study tried to obtain participants’ perspectives on 11 fixed barriers to knowledge sharing that were included in the questionnaire. The overall response of students was “moderately agree” on those barriers. Moreover, most students agreed that “too busy/lack of time” and “lack of relationship” are barriers to knowledge sharing (mean scores of 3.60 and 3.46, respectively). However, 199 (40%) participants did not perceive cultural diversity as a barrier to knowledge sharing. A lack of appreciation for knowledge sharing was recognized by 218 (43.9%) participants. Most respondents (378, 76.1%) believed that the fear of providing wrong information limited knowledge sharing (mean score of 3.95), which was the highest mean score. In addition, the statement “fearfulness of opposing viewpoints will insult others” was identified by 206 (41.4%) of students. On the other hand, 132 (26.6%) respondents disagreed with the statement that “students fear that others would perform better” would affect knowledge sharing. Furthermore, 193 (38.8%) of the respondents agreed with “do not know what to share” (mean score of 3.31). Another barrier to knowledge exchange was that they did not want to be perceived as “show-offs,” which was agreed upon by 234 (47.1%) participants. Moreover, almost half of the respondents (246, 49.5%) recognized “shyness to express personal ideas” as a barrier, which makes it a strong barrier. The majority of participants (309, 62.2%) agreed that “people only share with those who share with them” (mean score of 3.61) (Table 2).

**Table 2:** Barriers to knowledge sharing among medical students (n = 497). SD = standard deviation; n = total number of respondents (total number of participants = 497); N = total number of those who strongly agree, agree, are neutral, disagree, or strongly disagree

Statement	Strongly agree, N/n (%)	Agree, N/n (%)	Neutral, N/n (%)	Disagree, N/n (%)	Strongly disagree, N/n (%)	Mean	SD*
Too busy; lack of time	101/497 (20.3)	210/497 (42.3)	98/497 (19.7)	63/497 (12.7)	25/497 (5)	3.60	1.097
Lack of relationship	87/497 (17.5)	194/497 (39)	99/497 (19.9)	94/497 (18.9)	23/497 (4.6)	3.46	1.121
Lack of knowledge-sharing culture	69/497 (13.9)	115/497 (23.1)	114/497 (22.9)	157/497 (31.6)	42/497 (85)	3.02	1.201
Lack of appreciation of knowledge sharing	74/497 (14.9)	144/497 (29)	101/497 (20.3)	126/497 (25.4)	52/497 (10.5)	3.12	1.243
Afraid to provide the wrong information	175/497 (35.2)	203/497 (40.8)	58/497 (11.7)	41/497 (8.2)	20/497 (4)	3.95	1.077
Afraid that an opinion mismatch would offend others (including lecturers, tutors, etc.)	59/497 (11.9)	147/497 (29.6)	117/497 (23.5)	138/497 (27.8)	36/497 (7.25)	3.11	1.153
Afraid that others would perform better	74/497 (14.9)	112/497 (22.5)	69/497 (13.9)	132/497 (26.6)	110/497 (22.1)	2.81	1.393
Do not know what to share	54/497 (10.9)	193/497 (38.8)	129/497 (26)	96/497 (19.3)	25/497 (5)	3.31	1.059
Do not want to be perceived as a “show-off”	95/497 (19.1)	139/497 (28)	98/497 (19.7)	114/497 (22.9)	51/497 (10.3)	3.23	1.279
Shy to provide own opinions	78/497 (15.7)	168/497 (33.8)	91/497 (18.3)	117/497 (23.5)	43/497 (8.7)	3.24	1.221
People only share with those who share with them	101/497 (20.3)	208/497 (41.9)	94/497 (18.9)	79/497 (15.9)	15/497 (3)	3.61	1.071

Barriers to knowledge sharing in relation to gender

Female students’ opinions on the statements “lack of relationship” (mean of 3.66), “afraid to provide the wrong information” (mean of 4.11), “do not know what to share” (mean of 3.53), and “shyness to provide own opinions” (mean of 3.45) were significantly more dominant (p-values of 0.007, 0.020, 0.002, and 0.009, respectively) than male students’ opinions. There were small differences between male and female opinions on the other barriers but without any significant correlation, as demonstrated in Table 3.

**Table 3:** Barriers to knowledge sharing about gender. P-values were calculated using independent-samples t-test; * = significant p-value <0.05; M = mean; SD = standard deviation

Statement	Male	Female	P-value
	Mean ± SD	Mean ± SD	
Too busy; lack of time	3.54 ± 1.115	3.73 ± 1.050	0.069
Lack of relationship	3.36 ± 1.129	3.66 ± 1.082	0.007*
Lack of knowledge-sharing culture	3.07 ± 1.193	2.93 ± 1.216	0.205
Lack of appreciation of knowledge sharing	3.20 ± 1.214	2.98 ± 1.293	0.064
Afraid to provide the wrong information	3.87 ± 1.088	4.11 ± 1.040	0.020*
Afraid that an opinion mismatch would offend others (including lecturers, tutors, etc.)	3.06 ± 1.179	3.22 ± 1.091	0.150
Afraid that others would perform better	2.77 ± 1.356	2.91 ± 1.468	0.282
Do not know what to share	3.21 ± 1.035	3.53 ± 1.081	0.002*
Do not want to be perceived as a “show-off”	3.26 ± 1.263	3.17 ± 1.314	0.482
Shy to provide own opinions	3.15 ± 1.217	3.45 ± 1.207	0.009*
People only share with those who share with them	3.67 ± 1.053	3.48 ± 1.099	0.061

Barriers to knowledge sharing in relation to universities

KSU students showed more agreement with the statements “afraid to provide the wrong information” (mean of 4.05) and “afraid that an opinion mismatch would offend others (including lecturers, tutors, etc.)” (mean of 3.30) than students from other universities, but without any significant difference (p-values of 0.067 and 0.090, respectively). The weakest association was found among statements “do not know what to share,” “lack of appreciation of knowledge sharing,” and “afraid that others would perform better” (p-values of 0.741, 0.703, and 0.701, respectively). Generally, we could not find a significant relationship between barriers to knowledge sharing and universities (Table 4).

**Table 4:** Barriers to knowledge sharing in relation to universities. P-values were calculated using the one-way analysis of variance test. M = mean; SD = standard deviation; KSAU-HS = King Saud Bin Abdulaziz University for Health Sciences; KSU = King Saud University; IMSIU = Imam Mohammed bin Saud Islamic University

Statement	KSAU-HS	KSU	Alfaisal	IMSIU	P-value
	Mean ± SD	Mean ± SD	Mean ± SD	Mean ± SD	
Too busy; lack of time	3.59 ± 1.021	3.52 ± 1.227	3.93 ± 0.974	3.61 ± 1.108	0.186
Lack of relationship	3.48 ± 1.099	3.44 ± 1.157	3.64 ± 1.123	3.33 ± 1.1240	0.522
Lack of knowledge-sharing culture	2.98 ± 1.159	3.00 ± 1.215	3.07 ± 1.169	3.18 ± 1.324	0.616
Lack of appreciation of knowledge sharing	3.19 ± 1.208	3.04 ± 1.259	3.14 ± 1.287	3.08 ± 1.304	0.703
Afraid to provide the wrong information	4.00 ± 1.038	4.05 ± 0.988	3.75 ± 1.059	3.71 ± 1.315	0.067
Afraid that an opinion mismatch would offend others (including lecturers, tutors, etc.)	3.01 ± 1.170	3.30 ± 1.085	2.95 ± 1.011	3.13 ± 1.269	0.090
Afraid that others would perform better	2.80 ± 1.379	2.77 ± 1.380	3.05 ± 1.413	2.83 ± 1.464	0.701
Do not know what to share	3.34 ± 1.013	3.24 ± 1.091	3.27 ± 1.149	3.38 ± 1.095	0.741
Do not want to be perceived as a “show-off”	3.24 ± 1.296	3.12 ± 1.185	3.20 ± 1.424	3.39 ± 1.317	0.511
Shy to provide own opinions	3.32 ± 1.191	3.16 ± 1.279	3.05 ± 1.238	3.29 ± 1.187	0.408
People only share with those who share with them	3.69 ± 1.050	3.45 ± 1.090	3.55 ± 1.066	3.68 ± 1.086	0.177

Barriers to knowledge sharing in relation to the current year of study

Overall, our results revealed that there was no significant difference between participants’ opinions on barriers to knowledge sharing in relation to their current year of study. Perspectives on statements such as “lack of relationship” and “too busy/lack of time” as barriers to knowledge sharing were more noticeable among first-year students (mean of 3.63 and 3.77) without significant correlation (p-values of 0.072 and 0.201, respectively). However, second-year students revealed more agreement with the statement “shy to provide their own opinions” (mean of 3.33) without a significant difference (p-value of 0.117) (Table 5).

**Table 5:** Barriers to knowledge sharing in relation to the current year of study. P-values were calculated using the one-way analysis of variance test. M = mean; SD = standard deviation

Statement	First year	Second year	Third year	Fourth year	Fifth year	P-value
	Mean ± SD	Mean ± SD	Mean ± SD	Mean ± SD	Mean ± SD	
Too busy; lack of time	3.77 ± 1.067	3.49 ± 1.120	3.49 ± 1.155	3.62 ± 1.032	3.71 ± 0.991	0.201
Lack of relationship	3.63 ± 1.071	3.33 ± 1.092	3.47 ± 1.130	3.48 ± 1.119	3.09 ± 1.203	0.072
Lack of knowledge-sharing culture	3.06 ± 1.141	2.95 ± 1.250	3.03 ± 1.272	3.02 ± 1.140	3.02 ± 1.158	0.989
Lack of appreciation of knowledge sharing	2.93 ± 1.266	3.17 ± 1.212	3.23 ± 1.262	3.26 ± 1.222	2.93 ± 1.136	0.160
Afraid to provide the wrong information	3.85 ± 1.160	3.95 ± 1.170	4.09 ± 0.915	3.79 ± 1.191	3.93 ± 1.074	0.190
Afraid that an opinion mismatch would offend others (including lecturers, tutors, etc.)	3.07 ± 1.161	2.81 ± 1.203	3.24 ± 1.075	3.16 ± 1.167	3.02 ± 1.288	0.124
Afraid that others would perform better	2.89 ± 1.482	2.81 ± 1.401	2.84 ± 1.389	2.80 ± 1.364	2.56 ± 1.216	0.745
Do not know what to share	3.37 ± 1.032	3.38 ± 1.170	3.25 ± 1.030	3.32 ± 1.116	3.31 ± 1.019	0.868
Do not want to be perceived as a “show-off”	3.24 ± 1.400	3.17 ± 1.302	3.19 ± 1.207	3.38 ± 1.251	3.16 ± 1.261	0.804
Shy to provide own opinions	3.26 ± 1.174	3.33 ± 1.191	3.30 ± 1.190	3.27 ± 1.285	2.78 ± 1.347	0.117
People only share with those who share with them	3.50 ± 1.049	3.57 ± 1.146	3.66 ± 1.084	3.73 ± 1.000	3.49 ± 1.100	0.487

## Discussion

This survey aimed to assess the barriers to knowledge sharing among medical students at private and public medical colleges. Knowledge sharing has the potential to accelerate the process of innovation, productivity, and the ability to add value to products and services, resulting in the strategic development of companies [13]. Identifying knowledge-sharing barriers will assist organizations in implementing the appropriate interventions to enhance knowledge-sharing among their employees and increase their ability to take action to increase job value. This study explored students’ perspectives on 11 fixed barriers included in the questionnaire. Similar to another study, we found that statements such as “afraid to provide the wrong information,” “people only share with those who share with them,” and “too busy, lack of time” were the most identified barriers to knowledge sharing [7]. Another study showed different results about the same 11 barriers [8]. The statement “people only share with those who share with them” obtained the highest mean score (3.81). Moreover, most students agreed that a lack of relationships is a barrier to knowledge sharing. This was reinforced by prior findings that found that the main challenges preventing employees from sharing knowledge are knowledge hoarding, a lack of communication skills, and poor personal ties with other departments [14]. In this survey, most participants did not see a lack of knowledge-sharing culture as a deterrent. This contradicted another study conducted in Singapore, which identified a lack of a sharing culture as one of the key obstacles to information sharing [15]. However, a later study supported our results regarding lack of time and inadequate relationships. This could be explained by the level of trust between students, which may play a vital role in building relationships. It has been acknowledged that trust is at the core of knowledge exchange, suggesting that academics share more knowledge when they have a higher level of trust in one another [16]. A previous study discovered that barriers to knowledge sharing included a lack of time, fear or anxiety, ignorance of the advantages, low interaction between the knowledge sharer and the recipient, incompatibility of experience levels, poor verbal and written communication skills, demographic differences, low social interaction, and a lack of trust in people, which is in line with our results [7]. The variations between studies could be because of the differences in features of the study population, particularly their social, cultural, and educational backgrounds. Additionally, our results clarified that being female significantly affects opinions on barriers to knowledge sharing, whereas years of study and university do not. This finding agrees with a previous study from Nigeria, which concluded that gender significantly influences knowledge sharing while academic grades and faculty do not [17]. An earlier survey from Turkey supports this finding [18]. In contrast to our findings, a study conducted in Pakistan found that male and final-year students had more positive opinions than female and first-year students [8]. As an explanation for this, the high percentage of male students may prevent many girls from interacting. According to a study done in the United States, there is a correlation between the amount of knowledge shared and students’ grades, but no obvious link exists between the quality of knowledge shared and students’ grades [19,20].

This study has some limitations. First, the observational nature of our study may affect establishing a causal relationship. Second, due to self-reporting bias, participants in a survey study may misreport their attitudes and opinions to appear better. Third, this study was undertaken at a few Saudi universities in one city. Due to differences in social backgrounds, the findings of this study may not be applicable in other countries. Fourth, students from private universities were 44 out of 497, representing only 9% of the total population. Fifth, we could not make direct and official contact with the universities due to the COVID-19 pandemic, which prevented us from conducting a paper-based questionnaire. Lastly, we received 520 responses, which represents around 10% of the targeted population; however, the exact number of respondents who received the questionnaire could not be known, so we were not able to assess the response rate. On the other hand, this survey was the first to assess barriers to knowledge sharing among medical students in Saudi Arabia and was conducted in larger institutions.

## Conclusions

Students in Saudi Arabia identified significant barriers such as “lack of relationship,” “afraid to provide the wrong information,” “do not know what to share,” and “shy to provide own opinions” as factors that interfere with knowledge sharing. In addition, it was noted that the female gender strongly influenced judgment on knowledge-sharing barriers. The outcomes of this study could assist Saudi university authorities in designing and implementing an all-inclusive knowledge-sharing strategy for students. Further research is needed to corroborate our findings.
